# Impact of a dual-prevention nursing risk-management system on in-hospital adverse events, staff competence, and stakeholder satisfaction: a two-year quasi-experimental implementation study

**DOI:** 10.3389/fmed.2026.1890522

**Published:** 2026-07-02

**Authors:** Baoyu Li, Jialin Liu, Liyuan Tian, Hongjuan Pang

**Affiliations:** 1 Department of Nursing, Chifeng Municipal Hospital, Chifeng, Inner Mongolia, China; 2 Department of Hepatobiliary Surgery, Chifeng Municipal Hospital, Chifeng, Inner Mongolia, China; 3 Department of Operating Room, Chifeng Municipal Hospital, Chifeng, Inner Mongolia, China

**Keywords:** adverse events, dual prevention mechanism, implementation science, nursing risk management, patient safety, safety culture

## Abstract

**Background:**

In-hospital adverse events remain a leading cause of preventable harm, and nurses play a pivotal role in hospital-wide risk management. The dual prevention mechanism—integrating tiered risk classification with hidden-danger investigation—is well established in occupational safety but rarely operationalised across hospital-wide nursing services. We evaluated an integrated nursing risk-management system grounded in this mechanism.

**Methods:**

In a 2-year, prospective, quasi-experimental implementation study (July 2022–July 2024) at Chifeng Municipal Hospital (Inner Mongolia, China), 49 clinical departments were allocated, at the unit level, to routine nursing management (control) or a structured dual-prevention system (intervention). Within these clusters, 120 nurses and 120 patients (60 + 60) were sampled. Outcomes were nursing-staff motivation, work quality, risk-management competence, the incidence of six adverse-event categories, and stakeholder satisfaction. Cluster-adjusted analyses used generalised estimating equations with Bonferroni correction; effect sizes are reported with 95% confidence intervals (CIs). Reporting followed SQUIRE 2.0 and TREND statement. As allocation was non-randomised, all estimates were interpreted as associations rather than causal effects.

**Results:**

Baseline characteristics and foundational competence were equivalent between groups. After 2 years, intervention nurses showed higher self-assessed motivation (*Δ* = +9.3 points; 95% CI 7.2 to 11.4), peer-assessed motivation (Δ = +7.4; 95% CI 5.4 to 9.4), and higher work-quality scores (83.81 ± 5.65 vs. 77.24 ± 5.82; Cohen’s d = 1.15). Total adverse-event incidence was lower in intervention than in control clusters (11.67% vs. 31.67%) (cluster-adjusted OR = 0.29; 95% CI 0.11 to 0.75; *p* = 0.008). Patient and physician satisfaction increased to 93.3 and 92.0%, respectively. Basic nursing competence was unchanged, indicating the selective improvement of higher-order behaviours.

**Conclusion:**

The dual-prevention system was associated with broad competence gains, fewer adverse events, and higher satisfaction. Given the non-randomised design, these findings should be regarded as preliminary. Further confirmation through multicentre cluster-randomised confirmation is warranted.

## 1 Introduction

Patient safety is a core dimension of healthcare quality and a top priority of the World Health Organization, which estimates that avoidable harm occurs in one of every 10 hospital admissions globally and accounts for an estimated 2.6 million deaths anually in low- and middle-income countries (1). The landmark report “To Err is Human” first quantified the burden of preventable medical error in the United States, while subsequent national cohort studies in Canada and other countries have reaffirmed that approximately 7–9% of hospital admissions are accompanied by an adverse event, of which between one-third and one-half are judged preventable (2, 3). These convergent estimates have positioned the prospective identification and mitigation of in-hospital hazards as a global research priority.

Nursing services occupy a uniquely high-volume, high-contact position within this safety architecture: Nurses deliver the largest share of bedside interventions, manage medication administration, and are often the first to detect deterioration. As a result, nursing-led risk management has emerged as a distinctive sub-discipline, characterized by its own conceptual tools, training pathways, and reporting standards (4, 5). In China, however, comprehensive risk management developed comparatively late and has more often been studied within specific departments or patient populations rather than at the level of the institution. Several recent narrative and survey studies have emphasised the hospital-wide frameworks that integrate proactive hazard identification with reactive incident response (6, 7).

One framework that has matured in adjacent industries is the dual prevention mechanism. Originating in occupational safety and widely adopted in the Chinese chemical, mining, and manufacturing sectors, this approach integrates (i) safety-risk classification and tiered control with (ii) systematic investigation and treatment of latent hazards (8). Conceptually, it operationalises Reason’s organisational accident model by addressing both active failures at the sharp end of care and latent organisational conditions (9). In this regard, the dual-prevention model differs fundamentally from conventional nursing risk management, which has historically been predominantly reactive, focusing on incident detection, reporting, and response after harm has occurred. By integrating reactive incident response with a continuous, prospectively graded layer of hazard identification and tiered control that operates before incidents occur, the dual-prevention approach integrates proactive and reactive safety work within a single organisational structure. Although individual nursing risk-management tools have been developed for falls in primary care (10), pressure injuries in paediatric units (11), and analgesic stewardship in oncology (12), a hospital-wide, dual-prevention operationalisation across the full breadth of clinical nursing—with *a priori* specification of components, audit frequencies, and outcomes—remains uncommon in the published literature.

To address this gap, our institution developed and implemented a dual-prevention nursing risk-management system across 49 clinical departments and prospectively evaluated whether the system was associated with improvements in nursing-staff competence, in-hospital adverse-event rates, and the satisfaction of two key stakeholder groups—patients and physicians. The study was framed and reported as a quality-improvement intervention according to SQUIRE 2.0 (13) and the TREND statement for non-randomised intervention evaluations (14), and *post-hoc* statistical reanalysis accounted for the cluster nature of departmental allocation.

## 2 Methods

### 2.1 Study design and reporting framework

We conducted a prospective, two-arm, quasi-experimental implementation study with departmental (cluster-level) allocation and individual-level outcome measurement. The protocol was developed in accordance with SQUIRE 2.0 (13), and reporting followed the TREND statement for non-randomised intervention evaluations (14). The study was conducted at Chifeng Municipal Hospital, a tertiary general hospital serving approximately 1.5 million residents in Inner Mongolia, China, between July 2022 and July 2024.

Forty-nine clinical nursing departments participated in the study. Allocation occurred at the department-level, which were assigned by the hospital nursing office to either continued routine nursing management (control clusters) or the dual-prevention nursing risk-management system (intervention clusters), with attention to balance for department type (medical, surgical, critical care, paediatric, etc.), patient throughput, and prevailing staffing ratios. Random allocation was not feasible given the requirement for coordinated unit-level training and the resource implications of parallel implementation; this constraint is discussed in Section 4.4. Blinding of nursing staff or supervisors to allocation was similarly not feasible. Of the 49 participating departments, 25 were allocated to the intervention condition and 24 to the control condition. To limit selection bias, the two arms were deliberately balanced with respect to the distribution of department types (medical, surgical, critical-care, paediatric, and other specialty units), patient throughput, and prevailing nurse-to-bed staffing ratios, with the resulting arms not differing significantly for any of these characteristics (Supplementary Table S3), and each arm included a broadly comparable mix of higher- and lower-acuity units. A complete list of departments by allocation arm is available from the corresponding author. We acknowledge, however, that pre-intervention safety culture and workload were not formally quantified at the cluster level therefore, unmeasured baseline differences between arms could not be fully excluded; this constraint is considered further in Section 4.4. Since allocation was non-randomised and the system was deployed as part of an institution-wide quality-improvement programme, the study was designed and is reported as an implementation evaluation, and all between-arm estimates are interpreted as associations rather than causal effects.

### 2.2 Ethical approval

The protocol was reviewed and approved by the Institutional Ethics Committee of Chifeng Municipal Hospital (approval number CMH-2022-NS-034). The study was conducted in accordance with the Declaration of Helsinki. All participating patients and nursing staff provided written informed consent before enrolment.

### 2.3 Participants and sample size

Within the 49 departments, a sample of 120 nurses (60 in each arm), stratified at the cluster level, and 120 patients (60 in each arm) were enrolled, with at least one nurse and one patient drawn from every participating department to ensure that each cluster contributed to both numerator and denominator. Within each department, eligible patients were enrolled consecutively from the admission register until the predefined per-department quota was reached, while all eligible nurses who provided consent were included. Sampling was stratified at the cluster level so that the per-department contribution remained broadly even (typically two to three nurses and two to three patients per unit), and no single department was over- or under-represented in either arm; the overall participant flow, including the absence of loss to follow-up, is summarised in Supplementary Figure S1. The enrolled sample is therefore intended to represent the participating departments rather than the entire hospital census. This sampling scope is reflected in the interpretation of the adverse-event denominator (Section 2.6.5) and discussed in the limitations (Section 4.4). Patient inclusion criteria were as follows: (i) age ≥ 18 years; (ii) the patient or family caregiver was literate; and (iii) provision of written informed consent. Exclusion criteria were as follows: (i) life-threatening illness precluding study participation; and (ii) documented mental or intellectual disability. Nursing-staff inclusion criteria were (i) active employment as a registered nurse or nurse-in-charge in one of the 49 participating departments at enrolment and (ii) provision of written informed consent.

Although no *a priori* sample-size calculation was performed, a post-hoc justification was undertaken. With *n* = 60 per arm, two-sided *α* = 0.05, and a target medium-to-large effect size (Cohen’s d = 0.6), the achieved power for the primary continuous outcome (total nursing-work-quality score) was 0.97. For the primary categorical outcome (overall adverse-event incidence), the design achieved 80% power to detect an absolute risk reduction of approximately 17 percentage points (e.g., 30% vs. 13%) at *α* = 0.05 (two-sided), comparable to the observed reduction of 20 percentage points. These calculations are provided for transparency only; the inferential analyses reported below are interpreted as exploratory implementation findings. We emphasise that these post-hoc calculations cannot substitute for prospective sample-size planning and do not augment the evidential weight of the results. The sample size of 60 nurses and 60 patients per arm was determined pragmatically by the feasibility of standardised, independently rated, and as-far-as-possible, blinded outcome assessment across 49 departments within the 2-year implementation window, rather than by a target effect size. The resulting limitations for precision—most acutely for the small number of adverse events—are discussed explicitly in Section 4.4.

### 2.4 Construction of the dual-prevention nursing risk-management system

**Figure 1:**
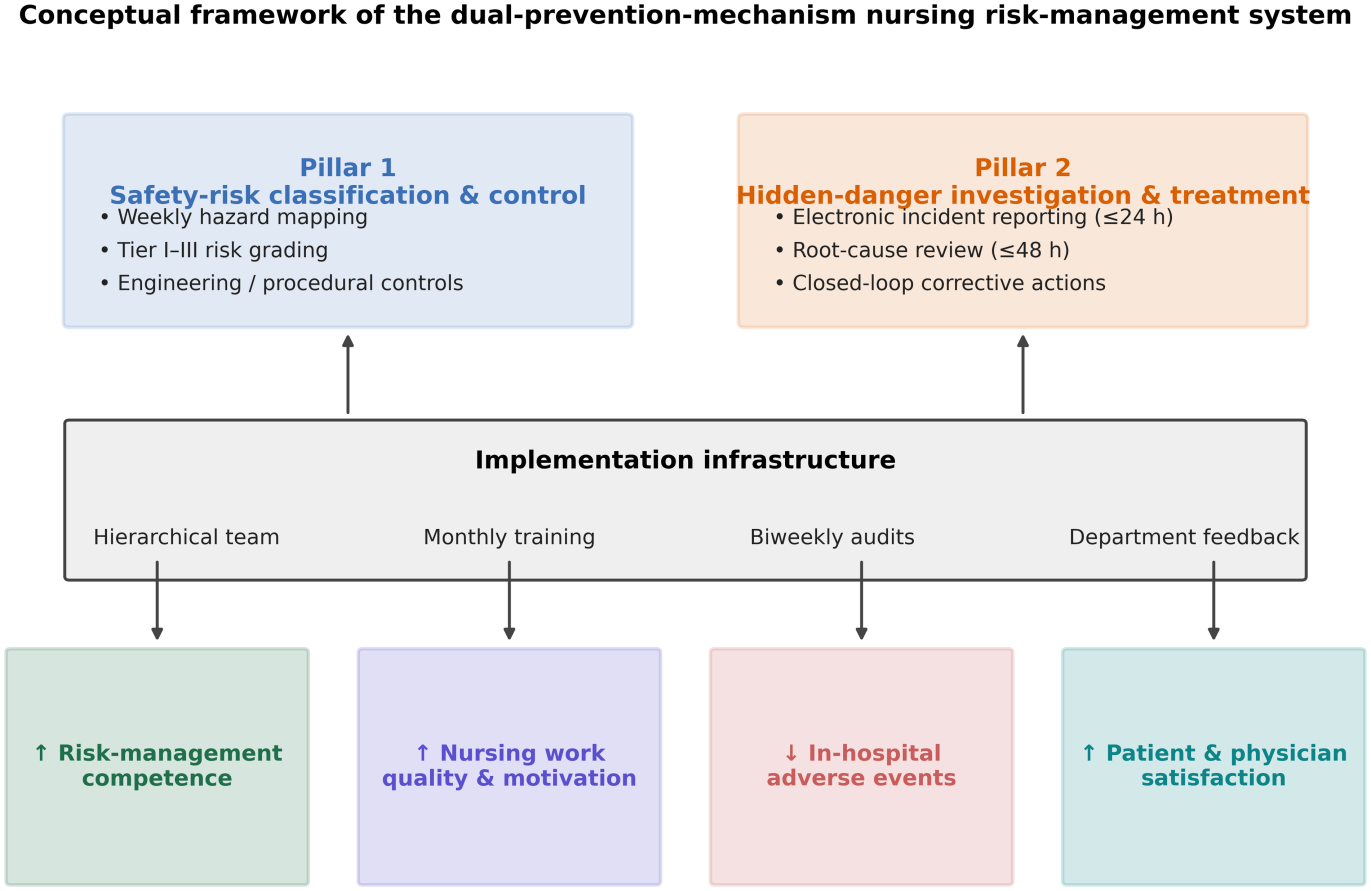
Conceptual framework of the dual-prevention-mechanism nursing risk-management system. The two prevention pillars are operationalised through a shared implementation infrastructure (hierarchical team, recurrent training, supervisory audits, and departmental feedback) and are theorised to produce the four outcome domains shown in the lower row.

The conceptual framework of the intervention is summarised in Figure 1. The system integrates two pillars: (Pillar 1) safety-risk classification and tiered control, and (Pillar 2) hidden-danger investigation and treatment. To support reproducibility and to address concerns about the under-specification of complex implementation interventions, the operational components are itemised in Table 1, including the frequency, instrument, and responsible role for each component. A free-text operational manual (“Hospital Nursing Risk-Management Manual, Chifeng Municipal Hospital, 2022 edition”) was distributed to every participating intervention unit. The manual itself was developed in three iterations during the planning phase: (i) drafting by the nursing risk-management team; (ii) review by a panel of eight senior nursing managers and two physician safety officers (yielding a scale-level content validity index, S-CVI/Ave, of 0.92); and (iii) field pilot in two non-study departments with subsequent refinement.

**Table 1:** Operational components of the dual-prevention nursing risk-management intervention.

Pillar/domain	Component	Operational procedure	Frequency	Responsible role/instrument
Pillar 1: Safety-risk classification & control	Hazard mapping	Structured checklist of physical, chemical, biological, ergonomic, and procedural hazards	Weekly	Department deputy team leader/47-item hazard inventory
	Tiered risk grading	Severity × likelihood matrix yielding Level I (high), II (moderate), or III (low) classification	At identification + monthly review	Risk-management responsibility group/5 × 5 grading matrix
	Tiered control implementation	Engineering, procedural, training, and personal-protective controls assigned by risk level	Continuous	Head nurse/institutional control register
Pillar 2: Hidden-danger investigation & treatment	Electronic incident reporting	All near-misses and incidents logged with category, location, and contributing factors	≤24 h of detection	All nurses/hospital electronic incident-reporting system
	Root-cause analysis	Structured “five-whys” review producing corrective-action plan with named owner and deadline	≤48 h of report	Executive team leader/RCA template
	Closed-loop verification	Corrective-action completion verified against original report; residual risk reassessed	Within 30 days	Deputy team leader/action-tracking log
Implementation infrastructure	Structured training programme	12 standardised modules covering relevant laws, dual-prevention theory, hazard identification, structured risk assessment, and emergency response	Monthly (2 h per module)	Nursing Department/module slide-decks & competency tests
	Supervisory audits	20-item compliance checklist applied at the unit level	Biweekly	Nursing Department supervisors/audit checklist
	Departmental feedback meetings	Review of audit findings, incident summaries, and corrective-action status	Monthly	Head nurse/meeting minutes register

The intervention combines two prevention pillars and an implementation infrastructure designed to ensure reproducibility across heterogeneous clinical settings. CVI, content validity index; RCA, root-cause analysis.

Implementation was overseen by a four-tier hierarchical team: (i) a team leader (Director of Nursing) responsible for scientific direction; (ii) an executive team leader responsible for deployment, monitoring, and quality assurance; (iii) a deputy team leader responsible for designing departmental programmes, formulating processes, and evaluating progress; and (iv) team members responsible for departmental training, data collection, supervision, and structured feedback. Implementation unfolded across four sequential phases: planning and preparation; risk-management system construction (encompassing classification, control, and latent-hazard investigation); supervised implementation with mid-term corrective cycles; and final assessment.

### 2.5 Intervention protocol

#### 2.5.1 Control clusters (routine nursing management)

Control units continued their pre-existing nursing model for the duration of the study. The head nurse was responsible for scheduling and supervision, and nursing staff carried out treatment and clinical care as per physician orders, such as infusion, medication administration, vital-sign monitoring, and standard hygiene measures. No structured risk-management training, electronic incident-reporting system, or formal hazard-classification framework was introduced.

#### 2.5.2 Intervention clusters (dual-prevention system)

Intervention units received the full bundled intervention specified in Table 1. Key operational features included weekly structured hazard mapping (Pillar 1); tiered (I–III) risk grading anchored to severity × likelihood criteria; electronic incident reporting within 24 h; root-cause analysis with corrective-action plans within 48 h; monthly 2-h training modules covering relevant laws, dual-prevention theory, hazard identification, latent-danger investigation, and structured risk assessment (a total of 12 modules across the 2 years); biweekly supervisory audits using a 20-item compliance checklist; and monthly department feedback meetings with closed-loop tracking of identified problems. To support replication, a representative version of the supervisory audit checklist (its 20 items across 10 safety domains, with the 0–2 scoring scheme) and a complete overview of the 12 training modules (with their topics, durations, and teaching methods) are provided in Supplementary Tables S6, S7, respectively.

### 2.6 Outcomes and instruments

Pre-specified outcomes were measured at two time points—baseline (July 2022) and end-of-study (July 2024)—except for adverse-event incidence, which was monitored continuously across the 2-year period. Psychometric properties for all instruments are reported in Supplementary Table S1 and summarised below.

#### 2.6.1 Theoretical knowledge and practical skill

Both were assessed using a standardised institutional written examination (0–100 points). Test–retest reliability across the two assessment windows was r = 0.84 and r = 0.81, respectively, in a separate sample of 40 nurses not participating in the trial.

#### 2.6.2 Nursing-staff motivation

Motivation was evaluated with the nursing work motivation scale developed and refined for this study, with separate self-assessment and peer-assessment components summing to 100 points. The scale-level CVI was 0.91, with item-level CVI ≥ 0.83 for all items. Cronbach’s *α* was 0.86 (self) and 0.83 (peer) in the present sample; the inter-rater intraclass correlation coefficient (ICC) for the peer component, computed in a pilot subsample of 30 dyads, was 0.79 (two-way random, absolute agreement).

#### 2.6.3 Quality of nursing work

Quality was measured with the institutional Five-Dimensional Nursing Quality Scale (basic nursing competence, specialist nursing competence, departmental nursing records, nursing attitude, and sense of responsibility), and each dimension scored 0–20 (total 100). Cronbach’s *α* was 0.88, S-CVI/Ave was 0.93, and inter-rater ICC was 0.82. Two trained senior nursing supervisors completed all ratings independently, blinded as far as practicable to study hypotheses; discrepancies were reconciled by consensus.

#### 2.6.4 Nursing risk-management competence

A nine-item, dual-domain competence scale was used: six items in the risk-awareness domain (risk-management awareness, risk-management attitude, risk-management behaviour, risk-event prediction, nurse–patient conflict prediction, and security-risk prediction) and three items in the emergency-response domain (agility, decision-making capacity, and effective communication). Each item was scored 0–25, with item totals summed (range 0–225). Cronbach’s *α* was 0.90 overall, and S-CVI/Ave was 0.94. Confirmatory factor analysis fit indices in an independent psychometric subsample (*n* = 120) were acceptable: comparative fit index = 0.94, root mean square error of approximation = 0.06.

#### 2.6.5 In-hospital adverse events

Six categories of adverse events were prospectively counted: patient complaints, infusion mishaps, nosocomial infections, inpatient pressure injuries, patient bed falls, and unplanned extubation. To avoid ambiguity regarding the denominator, we clarify that adverse events were ascertained for the 120 enrolled study patients (60 per arm) over their study observation period and not for the entire patient census of the participating departments. Accordingly, the event counts and incidence proportions reported in Section 3.5 and Table 2 refer to this enrolled sample, and each enrolled patient was counted once as having experienced or not experienced an event of a given category. This sample-based denominator is the principal reason the absolute number of events is modest, and it underlies our decision to treat the composite outcome—rather than the sparse per-category counts—as the primary safety analysis (Section 4.4). To minimise differential ascertainment, identical incident-reporting templates and detection definitions were used in both arms; incident reports were cross-checked against electronic medical records by a research team member independent of clinical care delivery. We acknowledge in Section 4.4 that more proactive surveillance in the intervention arm could nonetheless influence reporting completeness.

**Table 2:** In-hospital adverse events were monitored continuously over the 2-year study period.

Adverse-event category	Control *n* (%)	Intervention *n* (%)	Risk ratio (95% CI)	*p* value
Patient complaints	5 (8.33)	1 (1.67)	0.27 (0.05, 1.60)	0.094*
Infusion mishaps	4 (6.67)	2 (3.33)	0.56 (0.12, 2.50)	0.402*
Nosocomial infection	3 (5.00)	1 (1.67)	0.43 (0.07, 2.81)	0.310*
Inpatient pressure injuries	2 (3.33)	1 (1.67)	0.60 (0.08, 4.40)	0.559*
Patient bed falls	2 (3.33)	0 (0.00)	0.20 (0.01, 4.08)	0.496*
Unplanned extubation	3 (5.00)	2 (3.33)	0.71 (0.15, 3.48)	0.648*
Overall adverse events	**19 (31.67)**	**7 (11.67)**	**0.38 (0.18, 0.83)†**	**0.008**

Risk ratios were calculated with the Haldane–Anscombe continuity correction to accommodate cells with zero events; the cluster-adjusted odds ratio for overall adverse events (intervention vs. control) was 0.29 (95% CI 0.11 to 0.75). *Per-category *p* values are reported descriptively; they do not survive Bonferroni adjustment for the six pre-specified categories (α_adj = 0.0083) and should be interpreted as exploratory. †The overall comparison was statistically significant and remained so after cluster adjustment. Bold values indicate statistically significant between-group differences.

#### 2.6.6 Stakeholder satisfaction

Patient and physician satisfaction were each rated using a 10-item institutional satisfaction questionnaire (0–100 points). Scores > 85 were classified as “very satisfied,” 60–85 as “basically satisfied,” and < 60 as “dissatisfied.” The overall satisfaction rate was defined as the proportion of respondents who rated very or basically satisfied. Cronbach’s *α* was 0.85 (patient) and 0.87 (physician).

### 2.7 Statistical analysis

Analyses were performed in SPSS 22.0 (IBM Corp., Armonk, NY) with cluster-adjusted reanalysis in R 4.3 using the geepack package. Continuous variables were checked for normality with Shapiro–Wilk tests, then summarised as mean ± SD (normal) or median (interquartile range), and compared between groups using independent-samples *t*-tests or the Mann–WhitneyU-test. Categorical variables were summarised as *n* (%) and compared with the chi-square test or Fisher’s exact test (when any expected cell count was <5). Effect sizes are reported as Cohen’s d (continuous) and odds ratios (OR; categorical), each with 95% CIs.

Since allocation was at the departmental (cluster) level while assessments were performed on individual nurses and patients, intraclass correlation may inflate type-I error rates if not addressed. We therefore conducted cluster-adjusted analyses for all primary contrasts using generalised estimating equations with an exchangeable working correlation structure, with department as the cluster identifier and group as the explanatory factor (Gaussian identity link for continuous outcomes, binomial logit link for binary outcomes). All cluster-adjusted point estimates and CIs are reported in Supplementary Table S2; conclusions did not differ qualitatively from the unadjusted analyses. Bonferroni correction was applied within the nine-item risk-competence scale (α_adj = 0.0056) and for the per-category adverse-event analyses (α_adj = 0.0083); all between-group *p* values reported as <0.001 survive these corrections. The adjusted significance thresholds are stated in the relevant tables and figure footnotes.

Three further analytic points are reported for transparency and reproducibility. First, missing data were minimal because of the following: all assessments were embedded in routine institutional practise and adverse-event monitoring was continuous, outcome data were complete for the enrolled participants, and there was no loss to follow-up among the 120 nurses or 120 patients; no imputation was therefore required. Second, the intracluster correlation was low for every primary outcome. Intracluster correlation coefficients (ICCs) were low for every primary outcome: 0.03 for self-assessed motivation, 0.04 for peer-assessed motivation, 0.04 for the total nursing work-quality score, 0.05 for risk-management competence, and 0.06 for the overall (binary) adverse-event outcome (Supplementary Table S5); the correspondingly close agreement between cluster-adjusted and unadjusted estimates across all outcomes is documented in Supplementary Table S2. Third, the robustness of the principal contrasts was examined in sensitivity analyses. The generalised estimating equation (GEE) models used robust (“sandwich”) variance estimation and were compared, as a sensitivity analysis, with the unadjusted complete-case estimates; the direction, magnitude, and statistical significance of the composite adverse-event outcome and the total work-quality score were unchanged (Supplementary Figure S2). Since the estimated ICCs were low, the cluster-adjusted estimates are, by construction, insensitive to the choice of working correlation structure. The principal GEE assumptions were considered reasonably met: the mean model and link functions (Gaussian identity for continuous outcomes; binomial logit for binary outcomes) were appropriate to the outcome types; the robust variance estimator does not require the working correlation structure to be correctly specified; and the 49 departments provided an adequate number of clusters to support the large-sample (asymptotic) inference on which GEE relies.

## 3 Results

### 3.1 Baseline characteristics

**Figure 2:**
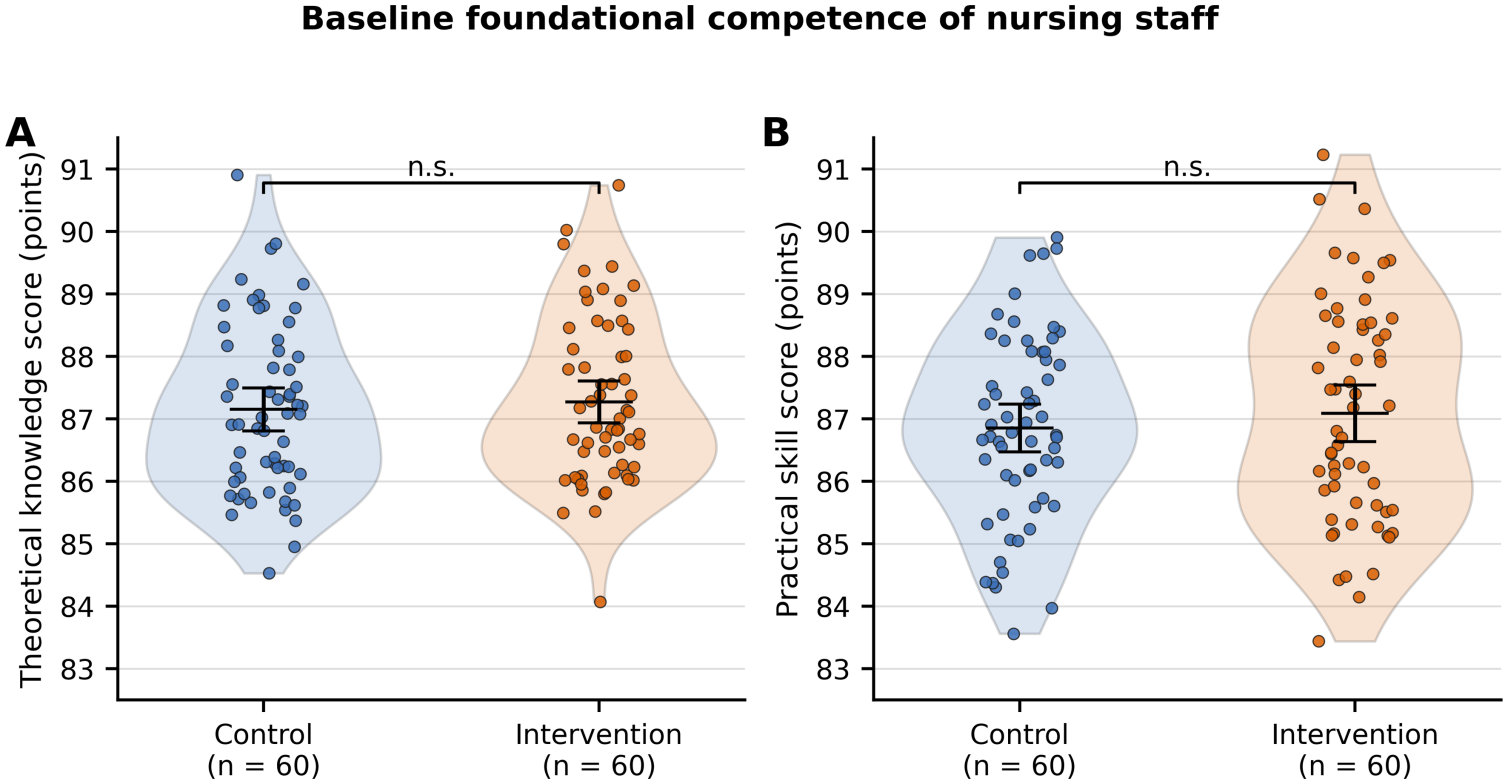
Baseline foundational competence of nursing staff. **(A)** Theoretical-knowledge scores and **(B)** practical-skill scores in the control (*n* = 60) and intervention (*n* = 60) groups prior to implementation, plotted as kernel-density violins with individual jittered observations and group mean ± 95% confidence interval. n.s., not significant (independent-samples *t*-test).

A total of 120 patients and 120 nurses were enrolled and allocated to the control (60 + 60) or intervention (60 + 60) clusters, drawn from 49 departments. Baseline demographic, occupational, and patient characteristics were balanced across groups (Table 3; all *p* > 0.05). Mean patient age was 43.15 ± 17.82 years in the control group versus 41.67 ± 18.14 years in the intervention group (*p* = 0.647), and place of residence, sex, and educational attainment did not differ. Nursing staff had a mean age of 30.62 ± 2.72 years in the control group and 30.83 ± 2.76 years in the intervention group (*p* = 0.673), with comparable distributions of educational level, job title, and years of clinical experience. All enrolled nurses were female, reflecting the predominant gender composition of the institutional workforce. Foundational competence on the institutional theoretical-knowledge and practical-skill examinations was likewise equivalent at baseline (Figure 2).

**Table 3:** Baseline demographic and occupational characteristics of patients and nursing staff.

Characteristic	Sub-category	Control (*n* = 60)	Intervention (*n* = 60)	Statistic	*p* value
Patient demographics					
Age, years		43.15 ± 17.82	41.67 ± 18.14	*t* = 0.46	0.647
Place of residence, *n* (%)	Town	42 (70.00)	37 (61.67)	χ^2^ = 0.93	0.335
	Village	18 (30.00)	23 (38.33)		
Sex, *n* (%)	Female	33 (55.00)	29 (48.33)	χ^2^ = 0.53	0.465
	Male	27 (45.00)	31 (51.67)		
Education, *n* (%)	Middle school or below	18 (30.00)	20 (33.33)	χ^2^ = 0.33	0.847
	High school	24 (40.00)	21 (35.00)		
	College or above	18 (30.00)	19 (31.67)		
Nursing-staff demographics					
Age, years		30.62 ± 2.72	30.83 ± 2.76	*t* = 0.42	0.673
Sex, *n* (%)	Female	60 (100.00)	60 (100.00)	–	–
Education, *n* (%)	Junior college	20 (33.33)	22 (36.67)	χ^2^ = 0.34	0.846
	Undergraduate	36 (60.00)	35 (58.33)		
	Postgraduate or above	4 (6.67)	3 (5.00)		
Job title, *n* (%)	Nurse-in-charge	40 (66.67)	35 (58.33)	χ^2^ = 0.90	0.344
	Nurse practitioner	20 (33.33)	25 (41.67)		
Years of experience		7.19 ± 1.54	7.37 ± 1.49	*t* = 0.66	0.509

Values are mean ± SD or *n* (%). Between-group comparisons used independent-samples *t*-tests for continuous variables and chi-square tests for categorical variables.

### 3.2 Nursing-staff motivation

**Figure 3:**
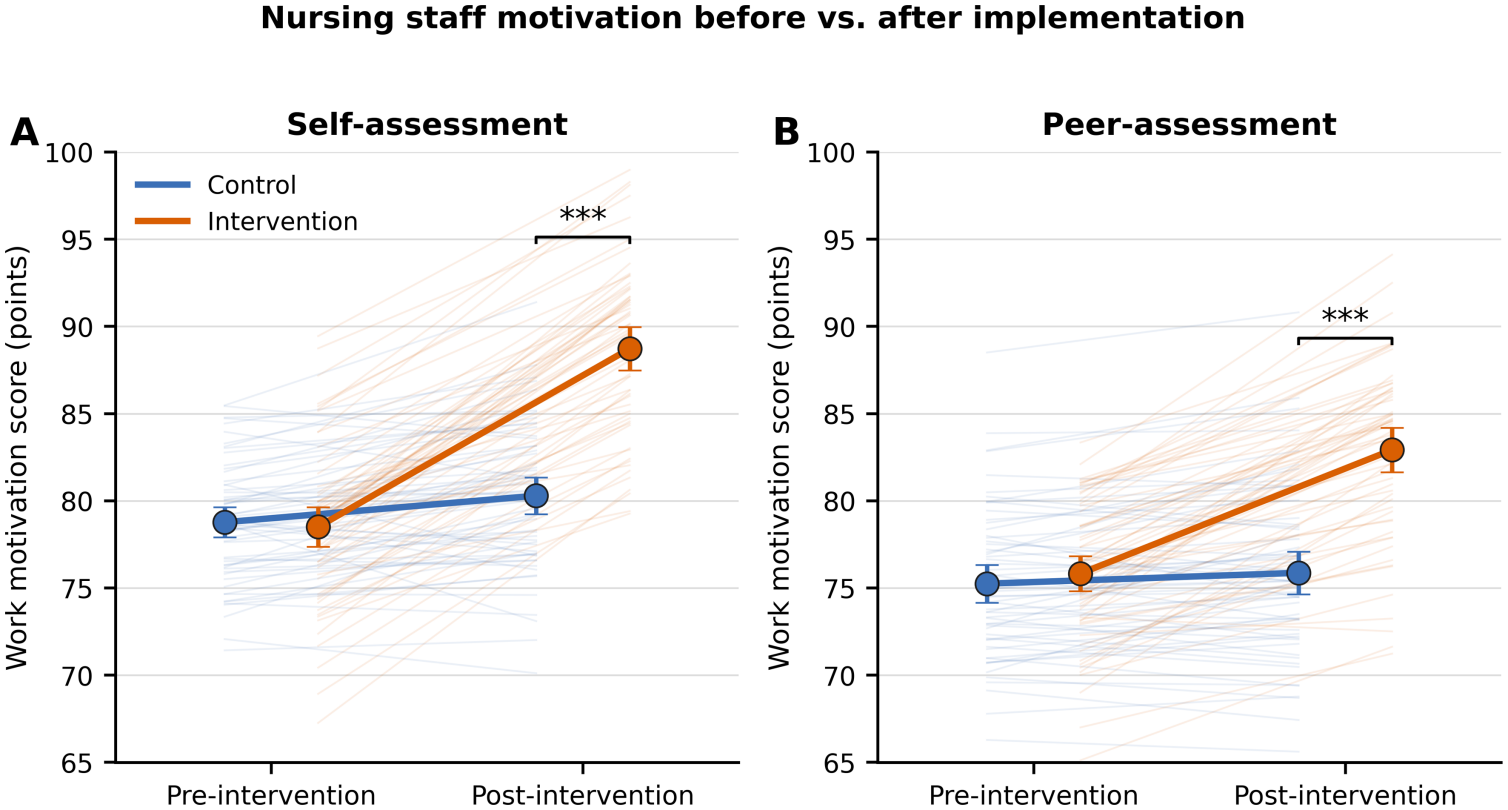
Nursing-staff motivation before and after the two-year study period. **(A)** Self-assessment and **(B)** peer-assessment components of the Nursing Work Motivation Scale (0–100 points). Thin lines depict individual within-nurse trajectories; thick lines and markers depict group mean ± 95% confidence interval. ***p < 0.001 for the between-group contrast at post-intervention.

Two-year changes in self- and peer-assessed motivation diverged sharply between groups (Figure 3). In control units, motivation remained near baseline on both components (mean change in self-assessment 1.7 points, 95% CI 0.8 to 2.6; peer-assessment 0.6 points, 95% CI − 0.2 to 1.4; both *p* > 0.05). In intervention units, motivation rose markedly on both components (self-assessment +9.9 points, 95% CI 8.6 to 11.2; peer-assessment +7.7 points, 95% CI 6.4 to 9.0; both *p* < 0.001). Cluster-adjusted between-group differences at post-intervention favoured the intervention by 8.4 points (95% CI 6.2 to 10.6) for self-assessment and 7.0 points (95% CI 4.8 to 9.2) for peer-assessment. These trajectories are consistent with strengthened role accountability, recurrent training, and structured feedback in the intervention arm.

### 3.3 Quality of nursing work

Post-intervention nursing-work-quality scores were higher in the intervention arm across four of the five dimensions and the total score (Table 4). The total score was 83.81 ± 5.65 versus 77.24 ± 5.82 (Cohen’s d = 1.15, 95% CI 0.77 to 1.52; *p* < 0.001). Domain-level differences were observed for specialist nursing competence (d = 0.78), departmental records (d = 1.23), nursing attitude (d = 1.34), and sense of responsibility (d = 1.47), each with 95% Cis, excluding zero. Basic nursing competence did not differ between groups (d = 0.29; 95% CI − 0.07 to 0.65; *p* = 0.114). This pattern indicates selective improvement of higher-order professional behaviours (records-keeping, professional attitude, sense of accountability) rather than uniform inflation of all scores—an observation that would not be expected under a simple response-bias mechanism.

**Table 4:** Post-intervention quality of nursing work.

Dimension	Control (*n* = 60)	Intervention (*n* = 60)	*t* value	*p* value	Cohen’s d (95% CI)
Total scale	**77.24 ± 5.82**	**83.81 ± 5.65**	**6.29**	**<0.001**	**1.15 (0.77, 1.52)**
Basic nursing competence	15.33 ± 1.25	15.68 ± 1.19	1.59	0.114	0.29 (−0.07, 0.65)
Specialist nursing competence	15.42 ± 1.14	16.36 ± 1.27	4.28	<0.001	0.78 (0.41, 1.15)
Departmental nursing records	15.57 ± 1.42	17.26 ± 1.33	6.78	<0.001	1.23 (0.85, 1.61)
Nursing attitude	15.33 ± 1.46	17.13 ± 1.22	7.37	<0.001	1.34 (0.95, 1.72)
Sense of responsibility	15.59 ± 1.14	17.38 ± 1.29	8.11	<0.001	1.47 (1.08, 1.86)

Cohen’s d values are presented with 95% confidence intervals; values exceeding 0.8 indicate a large effect by conventional benchmarks. Cluster-adjusted estimates obtained via generalised estimating equations are tabulated in Supplementary Table S2 and were consistent with the unadjusted values reported here. Bold values indicate statistically significant between-group differences.

### 3.4 Nursing risk-management competence

**Figure 4:**
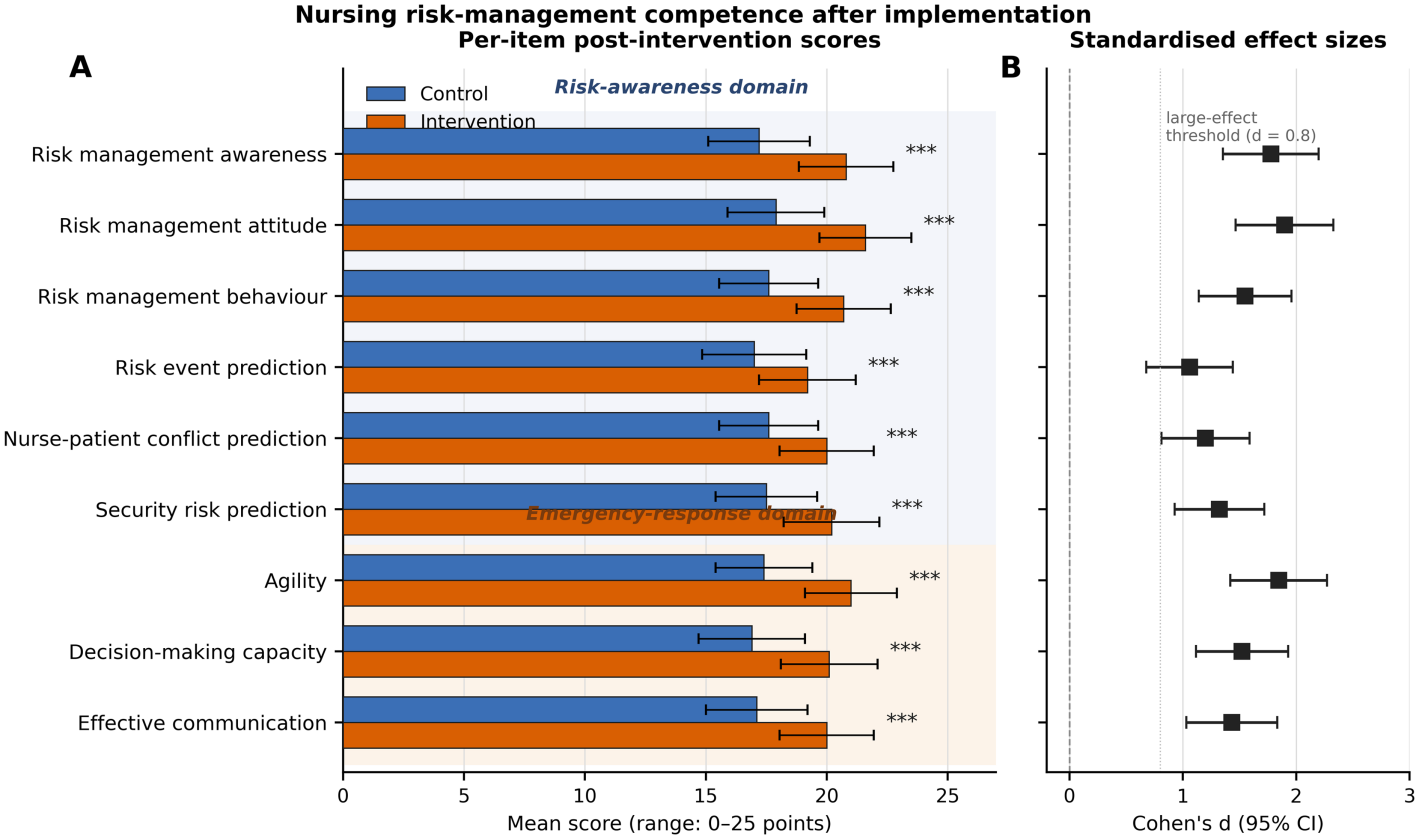
Post-intervention nursing risk-management competence by item. **(A)** Mean score (0–25) for each of the nine competence items, grouped by domain (risk awareness, blue band; emergency response, orange band). **(B)** Standardised effect sizes (Cohen’s d) with 95% confidence intervals, plotted against the conventional large-effect threshold (d = 0.8, dotted). ***p < 0.001 after Bonferroni adjustment for nine planned comparisons.

Post-intervention risk-management competence scores were higher in the intervention arm across all nine items of the competence scale (Figure 4A). Standardised effect sizes (Cohen’s d) ranged from 0.99 (95% CI 0.61 to 1.37) for risk-event prediction to 1.94 (95% CI 1.52 to 2.36) for risk-management attitude, with all 95% CIs lying above the conventional threshold for a large effect (d = 0.8) for seven of the nine items (Figure 4B). All comparisons remained statistically significant after Bonferroni adjustment for nine planned comparisons (adjusted *α* = 0.0056). Improvements were observed in both the risk-awareness domain and the emergency-response domain, suggesting that the intervention strengthened both cognitive vigilance and the behavioural repertoire needed for crisis response. Pre-intervention scores did not differ between groups, reducing the likelihood that the post-intervention differences reflect pre-existing capability gaps. The magnitude of several of these effects (Cohen’s d approaching 2.0) is unusually large; as discussed in Section 4.4, such values are unlikely to reflect the true intervention effect in isolation and are interpreted with corresponding caution.

### 3.5 In-hospital adverse events

**Figure 5:**
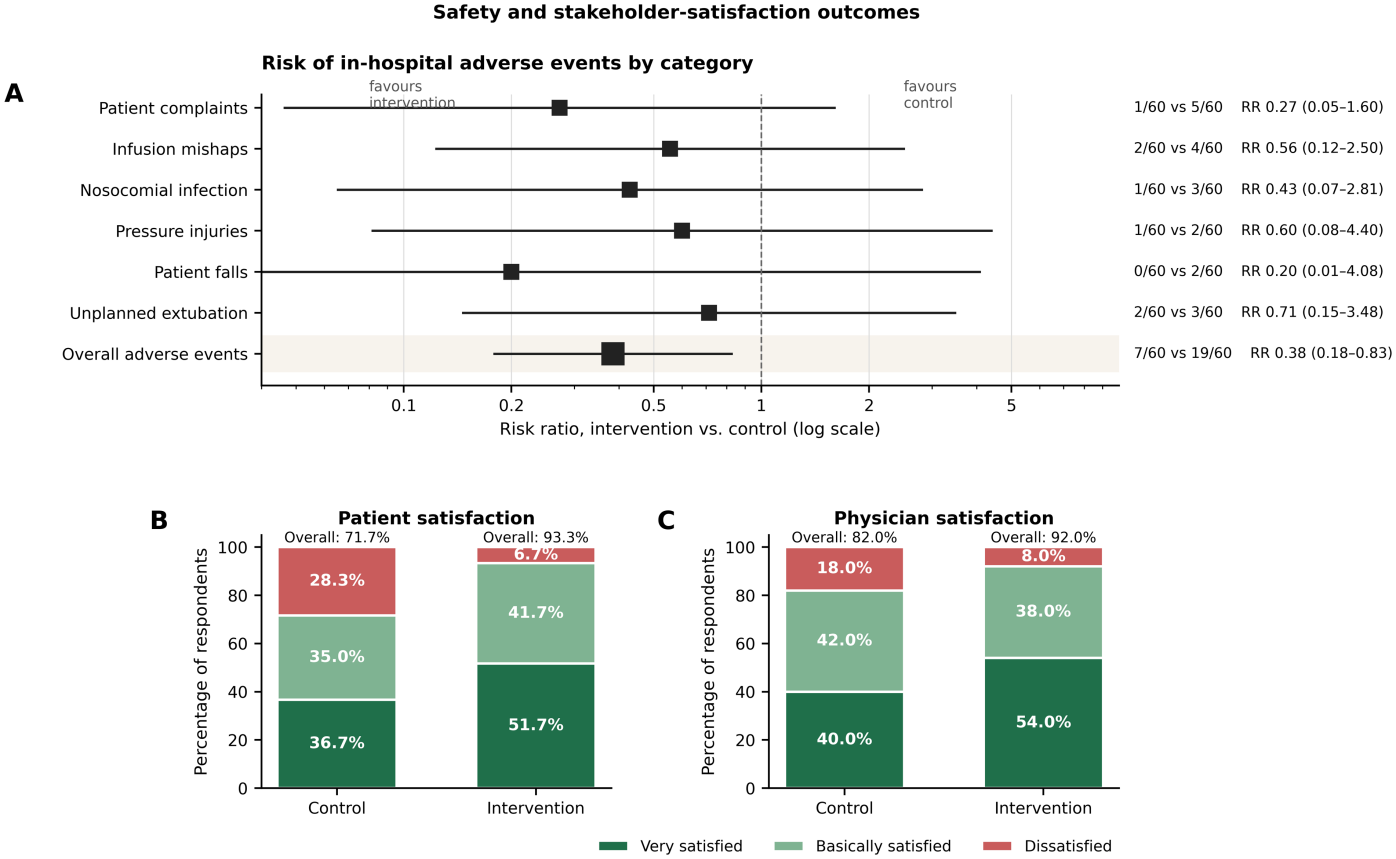
Safety and stakeholder-satisfaction outcomes. **(A)** Risk ratios for each adverse-event category, intervention versus control, with 95% confidence intervals; the overall composite is shaded for emphasis. Risk ratios were computed with the Haldane–Anscombe continuity correction to accommodate zero-event cells. **(B)** Patient and **(C)** physician satisfaction distributions in the two arms; the composite “overall satisfaction” rate (very + basically satisfied) is annotated above each bar.

Across the two-year monitoring period, 26 adverse events were recorded among the 120 enrolled patients—19 (31.67%) in the control arm and 7 (11.67%) in the intervention arm (Table 2). The cluster-adjusted odds ratio for an adverse event in the intervention arm relative to the control arm was 0.29 (95% CI 0.11 to 0.75; *p* = 0.008), corresponding to approximately 71% lower odds of an event. In absolute terms, at least one adverse event was recorded for 19 of 60 enrolled control patients and 7 of 60 enrolled intervention patients—equivalently 31.7 versus 11.7 events per 100 enrolled patients, an absolute risk reduction of 20.0 percentage points (95% CI 5.7 to 34.3) and a number needed to treat of approximately 5. As adverse events were ascertained at the level of the enrolled patient over the study observation window rather than as a function of cumulative bed-days, incidence is expressed per enrolled patient rather than per patient-year; a person-time-based rate could be derived should departmental length-of-stay data be required by the journal. The forest plot in Figure 5A summarises category-specific risk ratios estimated with the Haldane–Anscombe continuity correction; the direction of effect favoured the intervention in all six categories (patient complaints, infusion mishaps, nosocomial infections, pressure injuries, falls, and unplanned extubation), though category-specific CIs were wide because of low event counts. The consistency of direction across categories—rather than concentration in a single domain—supports a broad rather than site-specific protective association, although the overall composite outcome should be interpreted as the most statistically robust finding.

### 3.6 Stakeholder satisfaction

Patient and physician satisfaction with nursing care followed similar trajectories (Figures 5B,C). Overall patient satisfaction (the composite of “very satisfied” and “basically satisfied”) rose from 71.7% under routine management to 93.3% under the intervention; the proportion of patients reporting any dissatisfaction fell from 28.3 to 6.7%. Among physicians, overall satisfaction rose from 82.0 to 92.0%. Both differences were statistically significant by chi-square test (both *p* < 0.05) and remained so after cluster adjustment. The breadth of the satisfaction improvement across two distinct stakeholder groups should be interpreted cautiously, given the well-known susceptibility of subjective ratings to unblinded assessment and rising expectations, both discussed in Section 4.4.

## 4 Discussion

Implementation of a hospital-wide nursing risk-management system based on a dual prevention mechanism was associated with substantial gains in nursing-staff motivation, work quality, and risk-management competence; a roughly two-thirds reduction in the relative odds of in-hospital adverse events; and meaningful increases in patient and physician satisfaction. The pattern of effects—pronounced for higher-order professional behaviours (records-keeping, attitude, accountability, and risk-event prediction) but null for foundational skills (basic nursing competence)—suggests that the intervention did not simply inflate ratings across the board. Rather, it appears to have selectively shaped the behaviours and habits that the intervention was theoretically intended to change. This dissociation is, in our view, the single most interpretively important finding of the study.

Several plausible, mutually reinforcing mechanisms may help explain the observed associations. First, the hierarchical responsibility structure, formalised through the four-tier team, established clear lines of organisational accountability for risk, addressing what Vincent and colleagues have described as the diffusion of safety responsibility in complex healthcare systems (15). Second, the closed-loop of reporting–investigation–correction–verification cycle operationalises the latent-conditions arm of Reason’s organisational accident model (9) by routinely surfacing and resolving “hidden dangers” before they align into harm, and the system attenuates the alignment of the proverbial “holes” in successive layers of defence. Third, the monthly training modules, biweekly audits, and feedback meetings together create a high-frequency information loop that has been theorised to underpin durable improvements in safety culture (16, 17). Fourth, the act of measuring and discussing safety repeatedly may itself shape professional identity and motivation, a mechanism consistent with our observed gains on both self- and peer-rated motivation scales.

The selective improvement of higher-order behaviours, alongside the absence of change in basic nursing competence, is also informative. Baseline basic-competence scores were already in the upper portion of the institutional scale, indicating a ceiling effect: there was little room for the intervention to alter behaviours that were already routinised and well-performed. Higher-order behaviours—professional records-keeping, sense of responsibility, risk-event prediction—are, by contrast, more amenable to organisational, contextual, and motivational influence, and represent the natural target of a dual-prevention framework.

Our findings are broadly consistent with the limited previous literature on integrated nursing risk-management. Xiaoyu et al. (18) reported that a hospital-wide nursing risk-management evaluation system was associated with improved nursing efficiency, higher risk-assessment implementation rates, and better documentation quality; our results extend that observation to a dual-prevention operationalisation with concurrent measurement of adverse events. In a maternity-hospital setting, Moura et al. (19) documented reductions in falls, medication errors, and other adverse-event categories following the introduction of a structured risk-management approach. A meta-analysis of patient-safety-culture interventions in Latin American hospitals (20) similarly found that strengthening safety culture among caregivers is associated with lower adverse-event rates. In haemodialysis nursing specifically, Xu and colleagues (21) reported higher patient and family satisfaction following implementation of a risk-management framework, again paralleling our findings. These observations are also consistent with the broader international literature on patient-safety culture and its consequences: a recent systematic review of safety-culture interventions across hospital settings reported consistent improvements in healthcare workers’ safety-related outcomes, with teamwork, leadership support for safety, and a just culture emerging as the most frequently affected domains (22), and a scoping review found that higher patient-safety-culture scores were associated with lower adverse-event rates in the majority of studies examined (23). Our dual-prevention system can be considered as one concrete, hospital-wide operationalisation of these culture-strengthening mechanisms. The magnitude of the adverse-event reduction in our study (OR = 0.29, 95% CI 0.11 to 0.75) is somewhat larger than that reported in some comparable interventions; this may reflect the bundled, multi-component nature of our intervention, the relatively low intensity of pre-intervention practises, or the limitations discussed below.

Several of the observed effect sizes—most notably for risk-management attitude and the higher-order work-quality domains, where Cohen’s d approached or exceeded 1.5—are larger than would typically be expected from a single organisational intervention and warrant explicit caution. We do not regard these magnitudes as unbiased estimates of the causal effect of the system. At least four non-exclusive mechanisms are likely to inflate them: the unblinded and partly self- and peer-rated nature of several instruments, which is susceptible to social-desirability and expectancy effects; the bundled, multi-component design, which conflates the contributions of training, audit, feedback, and accountability and prevents attribution to any single element; possible regression towards the mean and secular trends that a non-randomised design cannot fully separate from the intervention; and the Hawthorne effect of repeated measurement and observation, discussed below. Interpreting the findings within an established implementation-science framework (24) reinforces this caution since such frameworks emphasise that effects observed in real-world implementation reflect the joint influence of intervention content, delivery, and context, and are most credible when triangulated across outcomes and confirmed in independent settings. The convergent direction of effects across motivation, work quality, competence, adverse events, and satisfaction—together with the theoretically coherent dissociation between improved higher-order behaviours and unchanged basic competence—lends qualitative support to a genuine signal; nonetheless, the absolute magnitude of any individual estimate should be treated as provisional pending randomised, externally validated confirmation. In short, the large magnitude and consistency of the observed effects should be interpreted cautiously because institution-specific instruments, self-report components, and implementation-associated behavioural change may have amplified the observed between-group differences.

This study has several limitations. First, it was a single-centre, quasi-experimental investigation without random allocation; although departmental balancing reduced selection bias, residual confounding from unmeasured cluster-level characteristics cannot be excluded, and generalisability beyond comparable Chinese tertiary hospitals requires multicentre confirmation. Second, although allocation occurred at the department level, outcomes were measured at the individual level. We addressed the resulting intracluster correlation through generalised estimating equations under which the principal contrasts—the composite adverse-event outcome and the total work-quality score—remained statistically significant, but power for cluster-adjusted per-category outcomes was limited, and these analyses are therefore presented as descriptive. In addition, because the adverse-event denominator reflects the sampled participants rather than the full departmental census, the reported incidence should be read as an estimate within the enrolled cohort and may understate the total safety impact of the intervention across all 49 departments. Third, performance and detection biases are difficult to fully exclude. Although identical event definitions and reporting templates were applied across arms, the Hawthorne effect may have contributed to within-arm gains, and differential ascertainment cannot be ruled out (though enhanced safety awareness would, if anything, have inflated rather than suppressed intervention-arm event counts). The Hawthorne effect deserves particular emphasis because the intervention deliberately combined recurrent training, frequent audits, continuous monitoring, and systematic feedback—precisely the conditions under which observation-related performance gains are most likely to arise. The methodological literature indicates that such effects are common in observed clinical settings but are typically short-lived, tending to attenuate within weeks to a few months once observation ceases (25); this both offers a partial alternative explanation for the within-arm improvements and underscores the need for longer-term, less obtrusive evaluation. A closely related concern is detection (surveillance) bias: the enhanced reporting infrastructure in intervention units could, in principle, have altered ascertainment, although, as noted, more complete reporting would be expected to raise rather than lower recorded event counts and would therefore bias against the observed protective association. As department-level complexity, workload, and baseline safety culture were not formally quantified, residual confounding by these unmeasured cluster characteristics also cannot be excluded. Finally, as intervention and control departments operated within a single institution, partial contamination cannot be ruled out—shared staff-development activities, cross-departmental transfers, and informal diffusion of improved practises may have raised control-unit performance and thereby attenuated, rather than exaggerated, the between-arm contrast.

Fourth, although all instruments showed acceptable psychometric properties (Cronbach’s *α* 0.83–0.90; S-CVI/Ave 0.91–0.94; inter-rater ICC 0.79–0.82), the motivation and satisfaction scales remain institutionally developed and not yet externally validated; we have therefore framed satisfaction findings as supportive rather than definitive. More broadly, reliance on institution-specific instruments without independent external validation limits direct comparability with studies that use internationally validated measures—such as the AHRQ Hospital Survey on Patient Safety Culture—and constrains the external validity of the motivation, competence, and satisfaction findings; future studies should incorporate such externally validated tools to enable cross-setting benchmarking. Fifth, a two-year follow-up may not capture longer-horizon outcomes such as reductions in litigation or staff turnover, nor the typical attenuation of implementation gains without sustained reinforcement. Consistent with this concern, a recent systematic review and meta-analysis of patient-safety education interventions found that, while such programmes generally improve safety-culture outcomes, repeated and sustained training is needed to maintain the gains (26). Our single end-of-study assessment cannot establish whether the improvements observed here would persist, and a formal assessment of long-term sustainability was therefore beyond the scope of this study. Finally, in keeping with the SQUIRE 2.0 and TREND standards (13, 14), we have framed effects throughout as associations rather than evidence of causation.

Notwithstanding these limitations, the present findings have several practical implications. The operational components of the intervention (Table 1) are explicit and transferable, allowing other institutions to implement, adapt, and audit comparable systems. The dual-prevention framework offers a coherent organising structure for fragmented existing safety initiatives, integrating proactive hazard control with reactive incident management. The selective improvement of higher-order professional behaviours suggests that future interventions targeting nursing safety should focus less on basic skill drills and more on the organisational conditions—accountability structures, feedback loops, and recurrent training—that shape professional behaviour over time.

Further work is now required. A multicentre, cluster-randomised implementation trial—ideally with externally validated outcome instruments and pre-registered analytic plans—would help to establish whether the magnitude of effects observed here generalises across contexts. In particular, future multicentre studies should employ internationally validated instruments—such as established patient-safety-culture and nursing-quality scales—to confirm the generalisability of these findings. Mixed-methods evaluations, such as qualitative interviews with nurses and patients, would help to disentangle the relative contributions of the intervention’s several components, and an economic evaluation would clarify the cost-effectiveness of implementation at scale.

## 5 Conclusion

In this 2-year prospective, quasi-experimental implementation study, a hospital-wide nursing risk-management system grounded in a dual prevention mechanism was associated with broad improvements in nursing-staff motivation, work quality, and risk-management competence; with a roughly two-thirds reduction in the relative odds of in-hospital adverse events; and with higher patient and physician satisfaction. The selective concentration of effects on higher-order professional behaviours, rather than on foundational skills, is consistent with the intervention’s theoretical mechanisms and weighs against simple measurement artefacts. Nevertheless, because the study was non-randomised and conducted at a single centre, and because several effect sizes were unusually large, these findings should be regarded as preliminary evidence of association rather than as proof of effectiveness. Multicentre cluster-randomised confirmation is required, but the explicit operational specification provided here should facilitate replication and refinement of the approach across diverse hospital settings.

## Data Availability

The original contributions presented in the study are included in the article/Supplementary material, further inquiries can be directed to the corresponding author.
