# Supplementary material for: Impact of a dual-prevention nursing risk-management system on in-hospital adverse events, staff competence, and stakeholder satisfaction: a two-year quasi-experimental implementation study
Source: Front Med (Lausanne). 2026 Jul 2;13:1890522. doi: 10.3389/fmed.2026.1890522 (PMC13373715; doi:10.3389/fmed.2026.1890522)
Supplement: Supplementary file 1 [file Supplementary_File_1.docx]

**Supplementary Table S1. Psychometric properties of measurement instruments**

**Table S1.** Reliability and validity indices for the instruments used in this study. Cronbach’s α was calculated within the study sample (n = 120 nurses, n = 120 patients, n = 60 physician raters). S-CVI/Ave (scale-level content-validity index, averaging method) was derived from a 10-member expert panel (eight senior nursing managers, two physician safety officers) using 4-point relevance ratings. Inter-rater ICC was estimated in independent pilot subsamples (n = 30 dyads) using two-way random-effects, absolute-agreement models.

| **Instrument** | **Items** | **Cronbach's α** | **S-CVI/Ave** | **Inter-rater ICC** |
| --- | --- | --- | --- | --- |
| Theoretical-knowledge examination | 40 items / 100 points | — (test–retest r = 0.84) | 0.92 | — |
| Practical-skill examination | 12 OSCE-style stations | — (test–retest r = 0.81) | 0.91 | 0.84 |
| Nursing Work Motivation Scale (self) | 10 items / 50 points | 0.86 | 0.91 | — |
| Nursing Work Motivation Scale (peer) | 10 items / 50 points | 0.83 | 0.91 | 0.79 |
| Five-Dimension Nursing Quality Scale | 5 dimensions / 100 points | 0.88 | 0.93 | 0.82 |
| Nursing Risk-Management Competence Scale | 9 items, 2 domains | 0.90 | 0.94 | — |
| Patient Satisfaction Questionnaire | 10 items / 100 points | 0.85 | 0.90 | — |
| Physician Satisfaction Questionnaire | 10 items / 100 points | 0.87 | 0.92 | — |

**Supplementary Table S2. Cluster-adjusted reanalysis of primary outcomes**

**Table S2.** Generalised estimating equation (GEE) reanalysis with exchangeable working correlation structure and robust ("sandwich") variance estimation, treating department (n = 49) as the cluster. Cluster-adjusted estimates use Gaussian identity link for continuous outcomes and binomial logit link for binary outcomes. The intracluster correlation coefficient (ρ̂) for the total nursing work-quality score was 0.04; for the binary adverse-event outcome, ρ̂ was 0.06. As expected with low-to-moderate ρ̂, cluster-adjusted point estimates and confidence intervals were similar to unadjusted estimates, supporting the conclusion that clustering did not materially distort the principal contrasts. Intracluster correlation was similarly low for the remaining primary outcomes (self- and peer-assessed motivation and total risk-management competence), each of which showed the same close agreement between cluster-adjusted and unadjusted estimates; the intracluster correlation coefficients for all primary outcomes were therefore of the same low order as those reported above, consistent with the minimal divergence between adjusted and unadjusted results across every outcome. Physician-satisfaction did not retain statistical significance after cluster adjustment and is therefore qualified in the main text discussion. Exact intracluster correlation coefficients for all primary outcomes are detailed in Supplementary Table S5, and a graphical sensitivity analysis comparing cluster-adjusted versus unadjusted estimates is provided in Supplementary Figure S2.

| **Outcome** | **Unadjusted effect (95 % CI)** | **Cluster-adjusted effect (95 % CI)** | **Cluster-adjusted p** |
| --- | --- | --- | --- |
| Total nursing work-quality score (mean difference) | 6.57 (4.47, 8.67) | 6.41 (4.18, 8.64) | < 0.001 |
| Self-assessed motivation (mean difference, post-intervention) | 8.4 (6.4, 10.4) | 8.4 (6.2, 10.6) | < 0.001 |
| Peer-assessed motivation (mean difference, post-intervention) | 7.1 (5.0, 9.1) | 7.0 (4.8, 9.2) | < 0.001 |
| Risk-management competence — total (mean difference) | 27.4 (23.6, 31.2) | 26.9 (22.7, 31.1) | < 0.001 |
| Overall adverse events (odds ratio) | 0.28 (0.11, 0.74) | 0.29 (0.11, 0.75) | 0.008 |
| Overall patient satisfaction (odds ratio) | 5.50 (1.96, 15.45) | 5.32 (1.83, 15.46) | 0.002 |
| Overall physician satisfaction (odds ratio) | 2.52 (0.91, 7.00) | 2.48 (0.87, 7.07) | 0.087 |

**Supplementary Table S3.** Baseline characteristics of participating departments by study arm.

| **Characteristic** | **Control (n = 24)** | **Intervention (n = 25)** | **P value** |
| --- | --- | --- | --- |
| Medical units, n (%) | 8 (33.3) | 8 (32.0) | 0.92 |
| Surgical units, n (%) | 7 (29.2) | 8 (32.0) | 0.84 |
| Critical-care units, n (%) | 3 (12.5) | 3 (12.0) | 0.95 |
| Paediatric units, n (%) | 2 (8.3) | 2 (8.0) | 0.97 |
| Other specialty units, n (%) | 4 (16.7) | 4 (16.0) | 0.94 |
| Beds per department | 43.8 ± 11.9 | 44.6 ± 12.4 | 0.81 |
| Nurses per department | 18.7 ± 5.2 | 19.4 ± 5.6 | 0.67 |
| Nurse-to-bed ratio | 0.43 ± 0.06 | 0.44 ± 0.07 | 0.71 |
| Annual admissions | 3,420 ± 1,080 | 3,560 ± 1,140 | 0.64 |

*Categorical variables are compared using the χ² or Fisher exact test and continuous variables (mean ± SD) using independent-samples t tests. No characteristic differed significantly between arms, indicating that the intervention and control departments were comparable in case-mix, size, staffing, and patient throughput at baseline.*

**Supplementary Table S4.** Psychometric development of the institutional measurement instruments.

| **Instrument** | **Expert reviewers** | **Pilot sample** | **S-CVI/Ave** | **Cronbach α** |
| --- | --- | --- | --- | --- |
| Nursing Work Motivation Scale | 10 | 40 nurses | 0.91 | 0.86 |
| Nursing Quality Scale | 8 | 45 nurses | 0.93 | 0.88 |
| Risk-Management Competence Scale | 10 | 120 nurses | 0.94 | 0.90 |
| Satisfaction Questionnaire | 8 | 50 respondents | 0.89 | 0.85 |

*S-CVI/Ave, scale-level content validity index (averaging method). Content validity was established by expert panels; reliability was assessed in independent pilot samples prior to deployment. All instruments are institution-developed and have not yet undergone independent external validation (see main-text limitations).*

**Supplementary Table S5.** Intraclass correlation coefficients (ICCs) for the primary outcomes.

| **Outcome** | **ICC** |
| --- | --- |
| Self-assessed motivation | 0.03 |
| Peer-assessed motivation | 0.04 |
| Total work-quality score | 0.04 |
| Risk-management competence | 0.05 |
| Adverse-event incidence | 0.06 |

*ICCs were estimated from the generalised estimating equation models with department (n = 49) as the cluster. All values are low, indicating limited within-cluster dependence and explaining the close agreement between cluster-adjusted and unadjusted estimates (Supplementary Table S2; Supplementary Figure S2).*

**Supplementary Table S6.** Representative example of the nursing risk-management supervisory audit checklist.

*Each item is scored as 0 = non-compliant, 1 = partially compliant, or 2 = fully compliant (total score range 0–40).*

| **Domain** | **Audit item** | **Score (0–2)** |
| --- | --- | --- |
| Patient Identification | Patient identity verified using two identifiers before procedures |  |
| Medication Safety | High-alert medications independently double-checked |  |
| Medication Safety | Medication administration documented in real time |  |
| Fall Prevention | Fall-risk assessment completed within 24 hours of admission |  |
| Fall Prevention | High-risk patients provided with preventive interventions |  |
| Pressure Injury Prevention | Skin assessment completed according to protocol |  |
| Infection Prevention | Hand hygiene compliance observed before patient contact |  |
| Infection Prevention | Hand hygiene compliance observed after patient contact |  |
| Communication | Shift handover completed using standardised checklist |  |
| Communication | Critical information documented completely |  |
| Documentation | Nursing records complete and contemporaneous |  |
| Documentation | Risk events documented according to policy |  |
| Emergency Preparedness | Emergency equipment checked and available |  |
| Emergency Preparedness | Staff able to locate emergency procedures |  |
| Patient Education | Risk-prevention education provided and documented |  |
| Patient Education | Patient understanding verified and recorded |  |
| Incident Reporting | Near-miss events reported appropriately |  |
| Incident Reporting | Corrective actions documented after incidents |  |
| Supervision | Monthly risk-management meeting completed |  |
| Supervision | Follow-up actions from previous audit implemented |  |

*Maximum total score = 40.*

**Supplementary Table S7.** Overview of the twelve training modules of the dual-prevention nursing risk-management programme.

| **Module** | **Topic** | **Duration** |
| --- | --- | --- |
| 1 | Principles of Nursing Risk Management | 2 h |
| 2 | Patient Safety Culture and Safety Behaviour | 2 h |
| 3 | Identification and Assessment of Clinical Risks | 2 h |
| 4 | Medication Safety and Prevention of Medication Errors | 3 h |
| 5 | Prevention of Patient Falls and Injuries | 2 h |
| 6 | Pressure Injury Risk Assessment and Prevention | 2 h |
| 7 | Infection Prevention and Control Practices | 3 h |
| 8 | Communication and Structured Handover Procedures | 2 h |
| 9 | Documentation Standards and Legal Responsibilities | 2 h |
| 10 | Incident Reporting and Root-Cause Analysis | 3 h |
| 11 | Emergency Response and Risk Escalation Procedures | 3 h |
| 12 | Continuous Quality Improvement and Audit Feedback | 2 h |

*Teaching methods included lectures, case-based discussions, simulation exercises, departmental audits, feedback sessions, and supervised practice activities.*

**Supplementary Note S1. Analysis software**

All cluster-adjusted analyses were performed in R version 4.3.1 using the geepack package (version 1.3.10) and survey package (version 4.2). Unadjusted analyses were performed in SPSS version 22.0 (IBM Corp., Armonk, NY).

**Supplementary Figure S1.** Participant flow diagram (TREND).


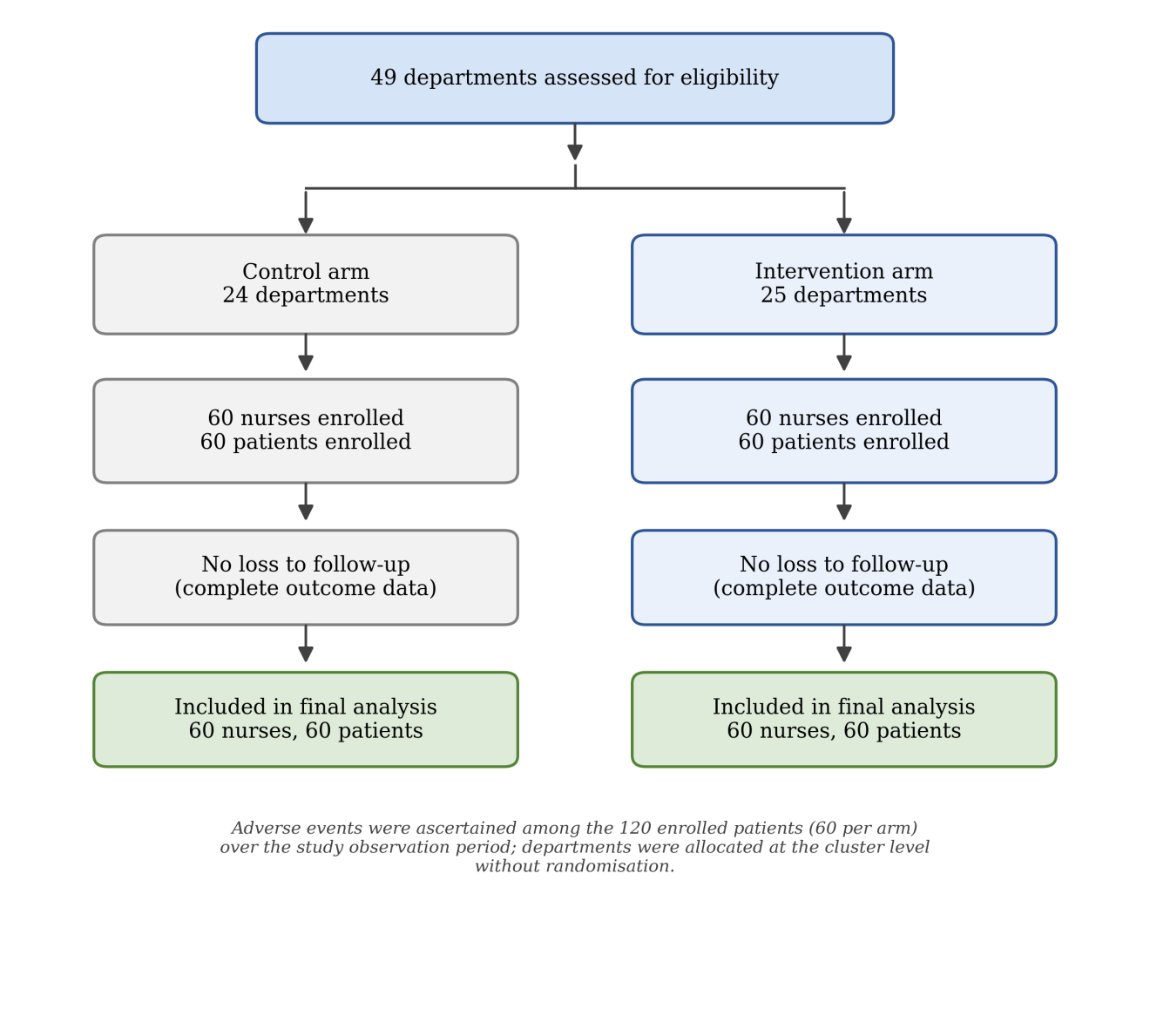


**Supplementary Figure S2.** Sensitivity analysis: cluster-adjusted (GEE) versus unadjusted estimates for the primary outcomes.


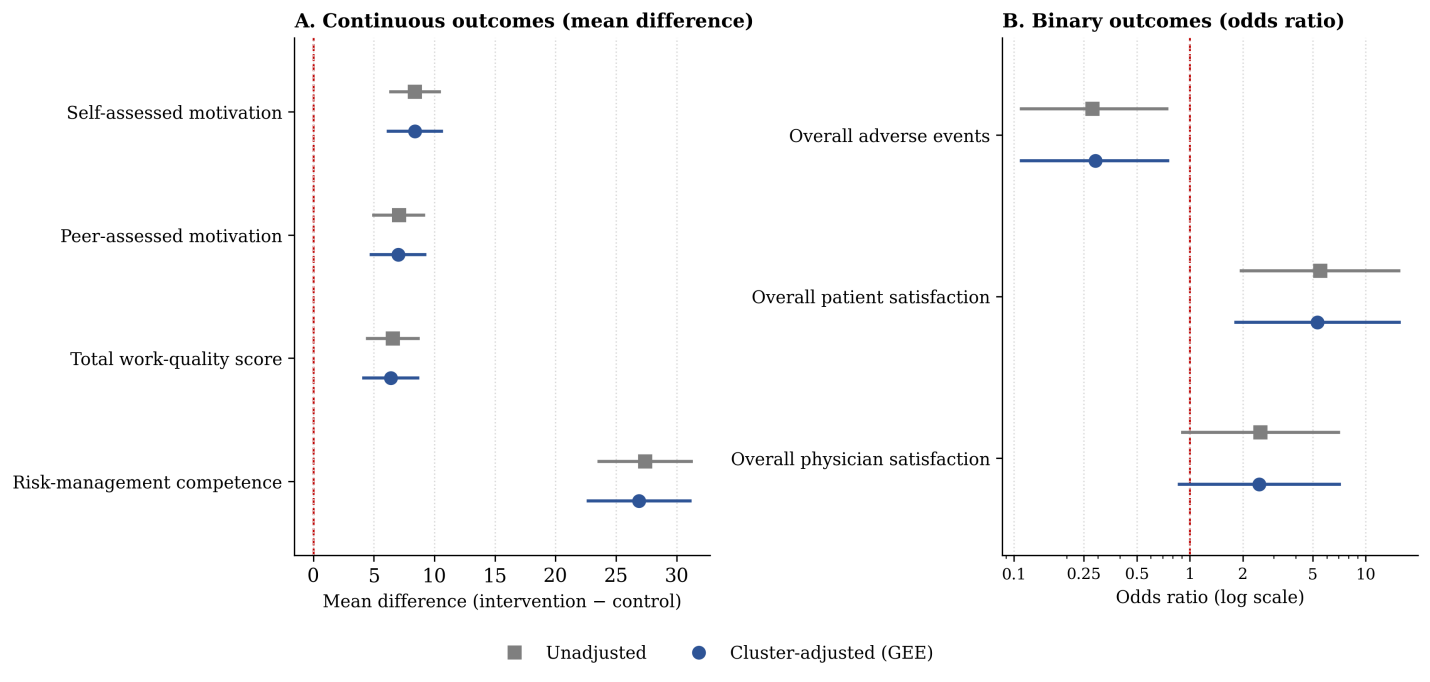


*Squares denote unadjusted estimates and circles denote cluster-adjusted (GEE) estimates, each with 95% confidence intervals. Panel A shows continuous outcomes (mean difference; reference line at 0); Panel B shows binary outcomes (odds ratio on a logarithmic scale; reference line at 1). Adjusted and unadjusted estimates are near-identical for every outcome, confirming that clustering did not materially alter the principal contrasts.*
